# Emergence of babesiosis in China-Myanmar border areas

**DOI:** 10.1186/s13071-015-0978-z

**Published:** 2015-07-25

**Authors:** Xia Zhou, Shang Xia, Shou-Qin Yin, Xiao-Nong Zhou

**Affiliations:** Department of Parasitology, Medical College of Soochow University, No.199 Renai Road, Suzhou, 215123 China; National Institute of Parasitic Diseases, Chinese Center for Disease Control and Prevention, WHO Collaborating Centre for Tropical Diseases; Key Laboratory of Parasite & Vector Biology, Ministry of Health, Shanghai, 200025 China; Tengchong Center for Disease Control and prevention, Tengchong, Yunnan province 679100 China

**Keywords:** Babesiosis, China-Myanmar border, Malaria, Syndemic, Emerging, Febrile case

## Abstract

E. Vannier and P. J. Krause presented an excellent article on “Babesiosis in China, an emerging threat” in the *Lancet Infectious Diseases* in December 2014, which updated research on human babesiosis in China. However, a neglected and emerging issue has not been mentioned in EV & PJK’s article, that is the co-infections with *B. microti* and *P. falciparum* parasites that exist in syndemic areas, spatially in the China-Myanmar border areas of Yunnan province, China. Therefore, two important issues are addressed in here, including (i) the new emerging infections with *Babesia* spp. which are normally ignored in malaria endemic areas due to similarities in pathogenic morphology and clinical symptoms, (ii) additional consideration on babesiosis rather than drug-resistant malaria when anti-malaria treatment for the febrile cases in clinics fails.

## To the Editor

**Fig. 1:**
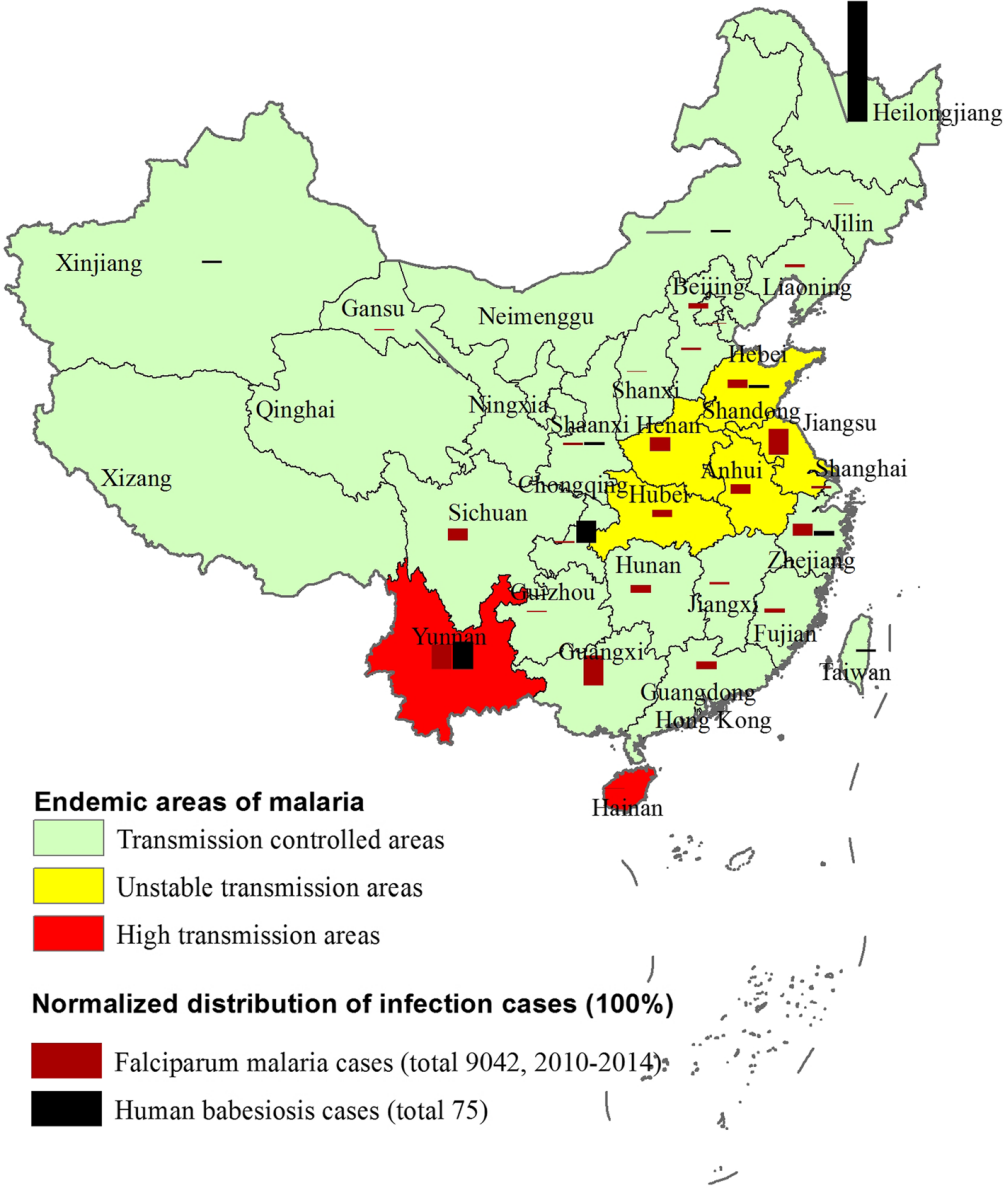
Syndemic areas of human babesiosis and falciparum malaria in China (High transmission areas of malaria including Yunnan province and Hainan province which are the syndemic areas of falciparum and vivax malaria (based on data in 2010). Unstable transmission areas including Jiangsu, Anhui, Shandong, Henan and Hubei province which are endemic areas of vivax malaria (based on data in 2010). Transmission controlled areas of malaria, other areas of China and all endemic areas of malaria were classified according to the National malaria control program ((2006-2015); Reported human babesiosis cases and falciparum malaria cases (2010-2014) were demonstrated by histograms)

E. Vannier and P. J. Krause presented excellent comments on “Babesiosis in China, an emerging threat” [1] which updated and replenished the review of “human babesiosis” [2] and reported human babesiosis cases in China [3–7]. Here, we would like to address a neglected and emerging issue that babesiosis has sometimes been misdiagnosed initially as malaria because of the similarities on morphology and symptoms between the two diseases. Especially, the intraerythrocytic ring form stages of *Babesia microti* can be misdiagnosed as ring forms of *Plasmodium falciparum* which are smaller than that of *P. viviax,* for the following three reasons. First, to date, little was known by medical doctors about the prevalence of *Babesia* spp. in malaria-epidemic areas, where misidentification as *Plasmodium* spp. is most probable. For instance, one molecular survey on febrile cases in malaria epidemic areas of southwestern China has shown that co-infections with *B. microti* and *P. falciparum* parasites exist along the China-Myanmar border in Yunnan province [7]. Secondly, misidentification of babesiosis as drug-resistant malaria, especially in syndemic areas, may occur due to some antimalarial drugs, such as chloroquine and mefloquine, having little effect on babesiosis [7, 8]. It was also reported that two patients, with a history of malaria, were tracked following treatment with artemether plus pyronaridine in the China-Myanmar border area [4]. Thirdly, infections with either *Babesia* spp*.* or *Plasmodium* spp*.* can cause severe diseases. The health burden imposed by both babesiosis and malaria could be substantial in these syndemic areas of China (Fig. 1), such as Yunnan province. Artemisinin-resistant *P. falciparum* malaria has been reported in Cambodia and the Thailand-Myanmar border [9]. Based on a small animal model*, B. microti* can be responsive to artemisinin derivatives [10]. However, in human babesiosis cases, there are still insufficient data to confirm that artemisinin derivatives are also effective to control infections of *Babesia*. Therefore, we strongly recommended that infection of *Babesia* should be excluded based on the results of laboratory-based molecular tests when the emergence of artemisinin-resistant is detected in the syndemic areas. It will help the standardization of babesiosis treatment using first choice drugs consisting of atovaquone and azithromycin and second choice drugs consisting of clindamycin and quinine [2]. Otherwise, the co-infections of human babesiosis and malaria would undoubtedly increase the pressure of artemisinin-resistance in malaria endemic areas because of the misuse of drugs.

To promote correct identification and effective control of babesiosis and malaria in syndemic areas, clinical doctors and public health workers should be aware of the following information: (i) how to apply the molecular technologies to clearly distinguish babesiosis from malaria in order to overcome the difficulties in microscopy diagnosis due to their similarities in morphology and clinical symptoms, and (ii) to consider human babesiosis rather than drug-resistant malaria when febrile cases in anti-malaria treatment fail.
